# Plant traits and vegetation data from climate warming experiments along an 1100 m elevation gradient in Gongga Mountains, China

**DOI:** 10.1038/s41597-020-0529-0

**Published:** 2020-06-19

**Authors:** Vigdis Vandvik, Aud H. Halbritter, Yan Yang, Hai He, Li Zhang, Alexander B. Brummer, Kari Klanderud, Brian S. Maitner, Sean T. Michaletz, Xiangyang Sun, Richard J. Telford, Genxu Wang, Inge H. J. Althuizen, Jonathan J. Henn, William Fernando Erazo Garcia, Ragnhild Gya, Francesca Jaroszynska, Blake L. Joyce, Rebecca Lehman, Michelangelo Sergio Moerland, Elisabeth Nesheim-Hauge, Linda Hovde Nordås, Ahui Peng, Claire Ponsac, Lorah Seltzer, Christien Steyn, Megan K. Sullivan, Jesslyn Tjendra, Yao Xiao, Xiaoxiang Zhao, Brian J. Enquist

**Affiliations:** 1 Department of Biological Sciences, University of Bergen, Bergen, Norway; 2 Bjerknes Centre for Climate Research, University of Bergen, Bergen, Norway; 3 Institute of Mountain Hazards and Environment, Chinese Academy of Sciences, Chengdu, China; 4 College of Life Sciences, Chongqing Normal University, Chongqing, China; 5 Department of Computational Medicine and Department of Ecology and Evolutionary Biology, University of California, Los Angeles, CA USA; 6 Faculty of Environmental Sciences and Natural Resource Management, Norwegian University of Life Sciences, Ås, Norway; 7 Department of Ecology and Evolutionary Biology, University of Arizona, Tucson, AZ USA; 8 Department of Botany and Biodiversity Research Centre, University of British Columbia, Vancouver, Canada; 9 Department of Integrative Biology, University of Wisconsin-Madison, WI, USA; 10 University Information Technology Services, University of Arizona, Tucson, AZ USA; 11 Department of Ecology and Evolutionary Biology, University of Arizona, Tucson, AZ USA; 12 Royal Botanic Gardens, Kew, Richmond, United Kingdom; 13 School of Biological Sciences, University of Reading, Reading, United Kingdom; 14 Departamento de Biología, Universidad de La Serena, La Serena, Chile; 15 Scientific Terrestrial Services, Johannesburg, South Africa; 16 Yale School of Forestry and Environmental Studies, New Haven, CT USA

**Keywords:** Natural variation in plants, Climate-change ecology, Community ecology

## Abstract

Functional trait data enhance climate change research by linking climate change, biodiversity response, and ecosystem functioning, and by enabling comparison between systems sharing few taxa. Across four sites along a 3000–4130 m a.s.l. gradient spanning 5.3 °C in growing season temperature in Mt. Gongga, Sichuan, China, we collected plant functional trait and vegetation data from control plots, open top chambers (OTCs), and reciprocally transplanted vegetation turfs. Over five years, we recorded vascular plant composition in 140 experimental treatment and control plots. We collected trait data associated with plant resource use, growth, and life history strategies (leaf area, leaf thickness, specific leaf area, leaf dry matter content, leaf C, N and P content and C and N isotopes) from local populations and from experimental treatments. The database consists of 6,671 plant records and 36,743 trait measurements (increasing the trait data coverage of the regional flora by 500%) covering 11 traits and 193 plant taxa (ca. 50% of which have no previous published trait data) across 37 families.

## Abstract (summary)

**Table:** 

Measurement(s)	leaf growth and development trait • Abundance • leaf area trait • leaf composition trait • leaf morphology trait • Vascular Plant • total biomass yield • climate
Technology Type(s)	balance • visual observation method • Scanner Device • leaf stoichiometry and isotope assays • weather station
Factor Type(s)	plants • temperature • plant functional traits (specific leaf area, leaf dry matter content, leaf carbon content, leaf nitrogen content, leaf size, leaf thickness) • type of treatment (e.g *in situ* warming experiments using an Open Top Chamber)
Sample Characteristic - Organism	Tracheophyta
Sample Characteristic - Environment	area of sedge- and forb-dominated herbaceous vegetation
Sample Characteristic - Location	Ganzi Tibetan Autonomous Prefecture

Machine-accessible metadata file describing the reported data: 10.6084/m9.figshare.12033786

## Background & Summary

Climate warming has wide-ranging impacts on biodiversity and functioning of alpine ecosystems, affecting phenology^1,2^, species ranges^3,4^, local plant abundance and biodiversity^5,6^, and ecosystem carbon, nutrient, and water fluxes^7^. However, there is also substantial variation between systems and regions in magnitude and directions of the response to a given climate change^8–10^. These context-dependencies limit our understanding of observed climate change impacts and our ability to forecast future trends in biodiversity and ecosystem functioning^11,12^. Comparisons across sites or regions, either through formalized replicated experiments or meta-analyses and other types of syntheses, are critically important to understand what drives the variation in biodiversity and ecosystem response to climate change.

Trait-based approaches offer opportunities for generalization and improved process-based understanding in global change ecology; and in particular for understanding drivers and consequences of context-dependencies in climate responses. Functional traits underlie variation among individuals in their ability to survive, reproduce, and function under different environmental conditions^13–15^. Because traits vary both within and between species, and can be affected by environment, evolutionary history and plasticity^16,17^, trait-based approaches offer great opportunity for improved understanding of the drivers, constraints and consequences of variability in biodiversity and ecosystem responses to climate change. In alpine regions, where temperature is a major limiting factor, traits associated with the leaf economics spectrum, a set of leaf traits that characterize individuals along a continuum from ‘fast’ to ‘slow’ photosynthetic and tissue turnover rates and life histories^18–20^, should be particularly relevant for responses to climatic warming. Functional traits thus improve our mechanistic understanding of species’ response to and functioning under climate change by providing a linkage between the phenotypes of individuals and the environment. An advantage of a trait-based approach is that trait measures offer a ‘common currency’ that facilitates comparison across regions and systems that may not share many taxa, as well as across experimental approaches.

Impacts of climate warming on plant communities are studied through four main empirical approaches. First, climate effects are assessed along natural elevation gradients, assuming a space-for-time substitution^21,22^. Second, ongoing climate change is studied through resampling of natural communities over time^6,23^. A third approach relies on on-site experimental warming, for example by Open Top Chambers (OTCs)^8,24^. A fourth approach is to transplant whole communities to warmer and/or cooler climates^25–27^. Each method has strengths and weaknesses, for example, gradient and resampling studies allow large-scale and long-term comparisons, OTCs experimentally expose intact extant communities to new climates, whereas community transplants manipulate both the biotic and the abiotic environment thus exploring the net effect of both direct and indirect climate change impacts^28,29^. Combining different approaches within a single study or system can be a powerful way to exploit these complementary strengths.

In this paper, we report a unique plant functional traits dataset collected from a combined OTC and transplant experiment along an 1100 m elevational gradient from 3000 to 4130 m above sea level (a.s.l.) in Mt. Gongga, Hengduan Mountains, Sichuan Province, China; referred to as «Mt. Gongga, China» below. Across four study sites we conducted an elevation gradient study, *in situ* warming experiments using OTCs, and reciprocal community transplantation experiments (warming and cooling) between all pairs of neighbouring sites and, as an extreme treatment, between the highest and lowest sites (extreme warming and cooling). In all three approaches, plant species richness, cover and associated climate data were measured each year from 2012 to 2016^29^. Plant functional traits of the vascular plant community were collected from the local grassland flora of each site in 2015 and 2016, and from the experimental treatments in 2016. The resulting datasets encompass vegetation and climate data and 36,743 trait measurements from 193 taxa, which extends existing trait data from the regional flora by ca. 100 additional species, and increases the number of unique trait measurements from this regional flora by 500%, according to the Botanical Information and Ecology Network (BIEN; http://bien.nceas.ucsb.edu/bien/) database^30^. These traits campaigns were conducted as part of two Plant Functional Traits Courses (PFTC1 and PFTC2) for international students in trait-based theory and methods (see also^31,32^). The data are comparable with data from later courses in Wayquecha, Peru (PFTC3 and 5) in 2018 and 2020 and Longyearbyen, Svalbard (PFTC4) in 2018 as well as with upcoming courses.

## Methods

### Research site selection and basic site information

**Fig. 1:**
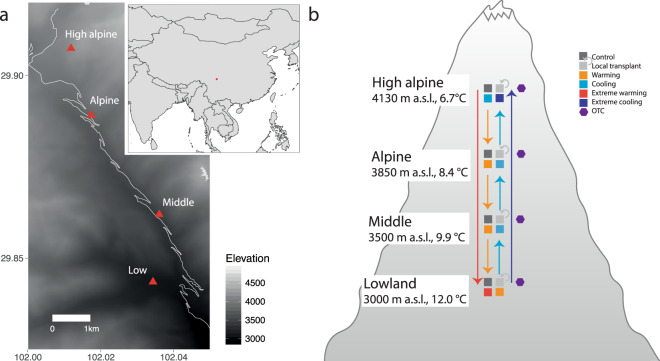
Location and study design of the Mt. Gongga climate change gradient and experimental studies. (**a**) Map of the study area with latitude, longitude, and elevation indicated as well as the location within China (insert), and (**b**) schematic representation of the study design of climate change experiments replicated across four sites along the 1100 m elevational gradient in Mt. Gongga, Sichuan Province, China. Each colour represents an experimental treatment; different kinds of turf transplants, Open Top Chambers (OTCs), and control plots (see legend). Arrows detail the turf transplant treatments between specific sites. At each site, all treatments are replicated in seven experimental blocks. Elevation and the mean temperature of the growing season (air temperature in the four warmest months per year, based on the 2 m climate station measurements, dataset v) are indicated for each sites.

The study was conducted in the Hengduan Mountains, Sichuan Province, China (Fig. 1). This area is on the south-eastern edge of the Tibetan Plateau, and the study sites are located in Kang-Ding Valley, on the northwest slopes of Mt. Gongga along a steep elevational gradient where the lower part is characterized by mixed coniferous-broadleaved forest, followed by belts of subalpine coniferous forest, sub-alpine meadows, subalpine shrub, and alpine meadows towards higher elevations and colder climates^33,34^.

In 2012, four study sites along the slope were chosen to reflect the major bioclimatic variation in southwest mountain grassland vegetation along an elevation gradient of 1100 meters; the Lowland site at 3000 m a.s.l, the Middle sites at 3500 m a.s.l., the Alpine site at 3850 m a.s.l., and the High Alpine site at 4130 m a.s.l. (Fig. 1, Table 1). All the sites were placed selectively in grasslands associated with mountain gray-brown soil originating from granite^35^. The sites were selected to represent the charactieristic grasslands at different elevations and hence climatic conditions, but to otherwise be as similar as possible with respect to environmental conditions, vegetation structure, plant community composition, slope, aspect, etc. The geographical distance between adjacent sites is on average ca 2 km.

**Table 1:** Basic site description from the Mt. Gongga climate gradient.

Site	Elevation (m a.s.l)	Latitude (°N)	Longitude (°E)	Summer temperature (°C)	GDD	Freezing days	Annual precipitation (mm)	Soil moisture (%)	Biomass
					>5 °C	<0 °C			(g/m^2^)
**High alpine**	4130	29.9074	102.0118	6.7	112	165	797	0.36	136.8 ± 8.8
**Alpine**	3850	29.8891	102.0173	8.4	130	148	821	0.38	248.8 ± 16.4
**Middle**	3500	29.8619	102.036	9.9	151	124	775	0.46	356.8 ± 25.2
**Lowland**	3000	29.8435	102.0343	12	181	90	784	0.38	271.6 ± 17.6

The four study sites with elevation, geographical coordinates, growing season mean temperature (June–August), based on data measured at 2 m above ground between 2012 and 2016 (dataset v), growing degree days > 5 °C (GDD), and freezing days with temperatures < 0 °C based on data from 2013 (dataset v), long term annual precipitation from WorldClim version 2.0 for 1970–2000^45^, soil moisture (June–August) measured at 5 cm below ground between 2012 and 2016 (dataset v) and above-ground standing biomass (dataset iii) in the four study sites along the slopes of Mt. Gongga, Sichuan Province, China.

At each site, we selected an experimental area within the grassland as homogeneous and representative of the grasslands at that elevation as possible. The experimental areas were placed on sloping ground, avoiding depressions and concave areas in the landscape and other features such as big rocks or formations that may affect microclimate, light conditions, hydrology and/or snowdrift. All sites were moderately grazed prior to the experiment by yak, sheep, cattle, goats, and/or horses, and the experimental areas were fenced and locked for the duration of the study to prevent grazing and human disturbance of the experimental infrastructure. The fenced area was mowed at the end of each growing season to mimic past grazing regimes and minimize fence effects.

### Experimental design

#### Block and plot setup

Within each of the four fenced experimental areas, we established seven replicate blocks, with a distance between the blocks ranging from four to six meters. Blocks were selectively placed in homogenous grassland, avoiding rocks, depressions, and other features as described above. Each block comprised five 25 cm × 25 cm plots, which were placed in a regular 3 rows x 2 columns grid (facing uphill) with 50 cm between adjacent plots. If a plot contained more than 10% bare rock, shrubs, or other non-grassland features, they were rejected or moved slightly to avoid these features. The plots were numbered by row from the uphill left corner of each block, permanently marked with plastic poles in each corner, onto which a standardized vegetation analysis frame could be mounted for vegetation (re)sampling. Within sites, the experimental area containing all experimental blocks is situated within a total area of ca. 75–200 m^2^.

In 2015, 73 additional 50 cm × 50 cm plots were set up within the fenced block areas in between the experimental blocks for biomass sampling (see dataset iii).

#### Transplant and Open Top Chamber) experiments

Within each of the seven blocks at each site, the pre-established and numbered plots were randomly designated to the following experimental treatments (with the specific treatments depending on the site, Fig. 1): (1) passive warming with an Open Top Chamber (OTC), (2) transplanting to a site one step warmer along the gradient (treatment ‘Warming’; from sites High alpine, Alpine, Middle), (3) transplanting to a site one step colder along the gradient (‘Cooling’; from sites Alpine, Middle, Lowland), (4) transplanting down the entire gradient (‘Extreme warming’; from site High alpine), and (5) up the entire gradient (‘Extreme cooling’, from site Lowland), (6) transplanting within blocks (to control for the transplanting itself)(‘Local transplant’; all sites), and (7) an untouched control plot (‘Control’; all sites). Thus, each OTC has a local unmanipulated control, and each transplanted turf has an “origin” site and a “destination” site, each with two types of controls, a local transplant and an untouched control plot (Fig. 1). The OTC chambers are hexagonal, 40 cm tall, with a distance between parallel sides of 106 cm at the base and 60 cm at the top. Snow cover is low and scattered at all sites, and the OTCs were left out year-round to achieve warming during the entire snow-free period. Because of the low snow cover, removing OTCs was also not necessary for their protection against snow damage.

We marked the upslope centre of each plot with a plastic flag to ensure that all plots were always analysed from the same direction, and that transplanted turfs retained their orientation relative to the slope and block layout at the destination site. For the transplant treatments, we used a knife to cut the turfs 2 cm outside the plot margins, giving turfs of 29 cm × 29 cm and to a depth of 20 cm, unless the organic soil was shallower, as was the case for some of the higher-elevation plots. After excavation, the turfs were packed into 29 cm × 29 cm waterproof boxes and transported to their respective destination sites within one or two days. To keep the transplant disturbance as similar as possible among treatments, the excavated control (‘home transplant’) turfs were also kept in boxes before they were put into their designated randomized destination plots within the site of origin.

The transplanted turfs were fitted into gaps created by excavating turfs at the destination site. Each block received one plot of each relevant treatment, resulting in five turfs per block, but with different specific treatments in the two middle sites and the extremes of the gradient (see Fig. 1). Transplanted turf positions were randomized within the destination blocks, while un-transplanted control and OTC warming plots - were not moved. Transplanted turfs were then carefully planted into their destination plots ensuring that the original orientation of the turf vs. the block slope was retained, the soil surface was level with the surrounding soil surface, and the edges of the excavated plot were in good contact with the sides of the gap. If necessary, loose soil was carefully removed from the underside of the turf, or local soil was added to the gap or around the edges to achieve this. This experimental design had a total of 140 plots. In 2014, two replicate blocks (10 plots) in the High alpine site were damaged by yaks, so having only five blocks in this site going forward.

### Species identification, taxonomy, and flora

All species sampled in the vegetation and functional trait datasets were identified in the field. Back at the field station, the identification of sampled whole plants or vouchers were checked by a taxonomic expert from the region using the Online Flora of China^36^. Specimens problematic to identify were brought back to the Chongqing Normal University for identification and deposition of vouchers by one of the co-authors (Prof. He). Forbs were identified to species level, whereas many of the graminoids were identified only to genus level, i.e., *Carex* spp., *Poa* spp., *Kobresia* spp., and *Festuca* spp., due to difficulties with identification of sterile graminoids as there may be undescribed taxa and as there are no keys for vegetative plants for this region (professor He, personal observation). All taxon names were standardized using the Taxonomic Name Resolution Service^37^.

### Dataset collection methods

#### Dataset (i): Plant community composition sampling

All vascular plant species in each plot were surveyed in 2012 (before treatment), and annually between 2013 and 2016. Each year, vegetation was surveyed during the peak of the growing season using a 25 cm × 25 cm frame overlain with a grid of 5 cm × 5 cm subplots. Subplots were numbered 1–25 starting from the up-slope left-hand corner of the plot, numbering the subplots from left to right by rows. We registered presence-absence of all species in each subplot, and estimated the percentage coverage of each species in the whole plot to the nearest 1%. Note that the total coverage in each plot can exceed 100, due to layering of the vegetation.

#### Dataset (ii): Vegetation height and structure sampling

Vegetation structure data for each plot was recorded between 2012 and 2016. Mean vegetation height and bryophyte depth was measured at five evenly spaced points per plot using a ruler. The total percent coverage of all vascular plants combined, was also recorded.

#### Dataset (iii): Biomass sampling

We measured the standing biomass per vascular plant species in the 73 additional 50 cm × 50 cm plots placed out in the general experimental area of each site in 2015. For each plot, species composition was registered, percentage cover of each species estimated using a 50 cm × 50 cm frame with 10 cm × 10 cm overlay, and the height measured at five positions using a ruler. The plot was then harvested at the ground level, sorted to species, and the biomass dried at 65 °C for 3 days before weighing to the nearest 0.0001 g. As the aboveground grass and forb biomass dies back each winter, standing biomass can be considered an approximation of aboveground net primary productivity in these grasslands.

#### Dataset (iv): Trait sampling and lab analyses

*Site-level sampling for leaf trait analyses*. We collected whole plants for leaf trait analyses from the common species in the plant community at each of the four sites in August 2015 and 2016. In 2015, we sampled as many species as possible from each of the four sites. In 2016, the collection was complemented to ensure that we had trait data from species making up at least 80% of the vegetation cover in all control plots at each site. The plants were collected outside of the experimental plots within a 50 m perimeter from the blocks, and we aimed to collect up to five individuals from each species in each site. To avoid repeated sampling from a single clone, we selected individuals that were visibly separated from other ramets of that species. The sampled plant individuals were labelled, put in plastic bags with moist paper towels, and stored in darkness at 4 °C until further processing. Processing was done as soon as possible, but due to unexpected high diversity in the grasslands and therefore many collected plants, some specimens were stored for longer than optimal, in 2015 for up to 4 days and in 2016 for up to 2 days. As a result, some of the site-level samples, especially from 2015, were less well hydrated/more wilted and/or generally in a poorer state than what is optimal for trait measurements (see below for discussion of data documentation and quality checking; note that the raw leaf scans are available for all leaves). Before processing, plant identification was checked vs. the Online Flora of China^36^. Up to three healthy, fully expanded leaves were then sampled from each individual. The leaves were cut off as close to the stem as possible, including the blade, petiole, and stipules when present. Further processing was completed within 24 hours (see below).

##### Experimental treatment-level leaf sampling for trait analyses

Ten of the most common species along the gradient were selected for more intensive sampling within the experimental treatments plots in August 2016. We targeted species (i) with broad distributions along the gradient, (ii) that were also locally frequent and therefore present in a number of turfs prior to the experiments, and (iii) that remained present in most treatments until the sampling year. This was to ensure sufficient replication within species across sites and treatments. Further, to prevent measuring individuals that had colonized the sampled plots during the course of the experiment, and therefore had not been subjected to the experimental treatments, we (iv) excluded species that spread fast clonally. The selected species for treatment-level analysis are *Artemisia flaccida*, *Epilobium fangii*, *Geranium pylzowianum*, *Hypericum wrightianum*, *Pedicularis davidii, Polygonum viviparum (Persicaria vivipara*), *Plantago asiatica*, *Potentilla leuconota*, *Veronica szechuanica*, and *Viola biflora* var. *rockiana*. Of these species, *P. leuconota* and *V. szechuanica* were present across the whole elevation gradient. All other species were sampled from all treatments at sites where they are naturally occurring, including from transplanted turfs outside of the natural elevation range. Because many species in this system display at least some clonal reproduction, distinguishing genetic individuals is impossible without destructive sampling, and we therefore work at the ramet level, following^38^. For all sites and selected species, we collected up to five healthy, fully expanded leaves from up to five ramets in all experimental plots (i.e., control, locally transplanted control, moderate and extreme warmed and cooled transplants, open top chamber) where that species occurred. The leaves were cut off as close to the stem as possible, including the blade, petiole, and stipules when present. The leaves were labelled, put in plastic bags with moist paper towels, and stored dark at 4 °C and further processed within 24 hours of collection.

##### Plant functional trait measurements

We measured 11 leaf functional traits that are related to potential physiological growth rates and environmental tolerance of plants, following the standardized protocols in Pérez-Harguindeguy *et al*.^39^: leaf area (LA, cm^2^), leaf thickness (LT, mm), leaf dry matter content (LDMC, g/g), specific leaf area (SLA, cm^2^/g), carbon (C, %), nitrogen (N, %), phosphorus (P, %), carbon nitrogen ratio (C:N), nitrogen phosphorus ratio (N:P), carbon^13^ isotope ratio (δ^13^C, ‰), and nitrogen^15^ isotope ratio (δ^15^N, ‰).

Initial leaf processing was done at the field station of the Institute for Mountain Hazards and Environment (IMHE) in Moxi, Sichuan. Processing was done in the following steps:

1. **Leaf area**. Leaves (including blade, petiole, and stipules when present) were carefully patted dry with paper towels, flattened (folded out to their maximum area) and scanned using a Canon LiDE 220 flatbed scanner at 300 dpi. Care was taken that no leaf parts were overlapping on the scanner, and naturally overlapping parts of lamina (e.g., as is the case in some compound leaves) were cut off and placed next to each other on the scanner to obtain the full leaf area. Leaves that could not be flattened because they are narrow and grow naturally folded (e.g., some *Festuca sp*.) were scanned as they were, thereafter the area was multiplied by 2 during data processing. Any dark edges on the scans were manually cropped. Leaf area was calculated using ImageJ^40^ and the LeafArea package^41^.
2. **Leaf wet mass**. Each leaf (including blade, petiole, and stipules when present) was weighted to the nearest 0.001 g to assess fresh mass.
3. **Leaf thickness**. Leaf thickness was measured at three locations on each leaf blade with a digital calliper (Micromar 40 EWR, Mahr) and the average was calculated for further analysis. When possible, the three measurements were taken on the middle vein of the leaf and on lamina with and without veins. The petiole or stipule thickness is not measured.
4. **Leaf dry mass**. Leaves (including blade, petiole, and stipules when present) were then dried for a minimum of 72 hours at 65 °C before dry mass was measured to the nearest 0.0001 g.
5. **Leaf stoichiometry and isotopes**. A subset of leaves (n = 576; 265 from the gradient sampling and 311 from the experiments) were transported to the University of Arizona for leaf stoichiometry and isotope assays (P, N, C, δ^15^N, and δ^13^C). The leaves were stored in a drying oven at 65 °C before shipping and processing. Each leaf (including blade, petiole, and stipules when present) was ground into a fine homogenous powder. Total phosphorus concentration was determined using persulfate oxidation followed by the acid molybdate technique (APHA 1992) and phosphorus concentration was then measured colorimetrically with a spectrophotometer (ThermoScientific Genesys20, USA). Nitrogen, carbon, stable nitrogen (δ^15^N) and carbon (δ^13^C) isotopes were measured by the Department of Geosciences Environmental Isotope Laboratory at the University of Arizona on a continuous-flow gas-ratio mass spectrometer (Finnigan Delta PlusXL) coupled to an elemental analyzer (Costech). Samples of 1.0 ± 0.2 mg were combusted in the elemental analyser. Standardization is based on acetanilide for elemental concentration, NBS-22 and USGS-24 for δ^13^C, and IAEA-N-1 and IAEA-N-2 for δ15N. Precision is at least ± 0.2 for δ15N (1 s), based on repeated internal standards. Ratios between C:N and N:P were calculated and used in the analysis.

#### Datasets (v-vii) – climate data

Climate stations (U30-NRC, Tempcon Instrumentation LTD, UK) at each site recorded precipitation, air temperature and humidity at height of 2 m above ground, and soil moisture were measured at depths of 0 cm, 5 cm and 20 cm below ground in 10 min intervals, starting in September 2012 and continuing during the whole study period. The same measurements were conducted inside OTCs except that here air temperature was measured at 20 cm above ground. Additionally, iButtons (iButtonLink Technologies, USA) were installed at 5 cm below ground, ground level, and 30 cm above ground inside and outside OTCs in each site along the gradient from June–August 2017, at 10 min intervals. Finally, TomsT TMS-4 loggers^42^ were installed inside and outside OTCs at the High alpine site from September to November 2019. These measure temperatures at 15 cm above ground, ground level, and 6 cm below ground, and soil moisture, at 15 min intervals.

## Data Records

**Fig. 2:**
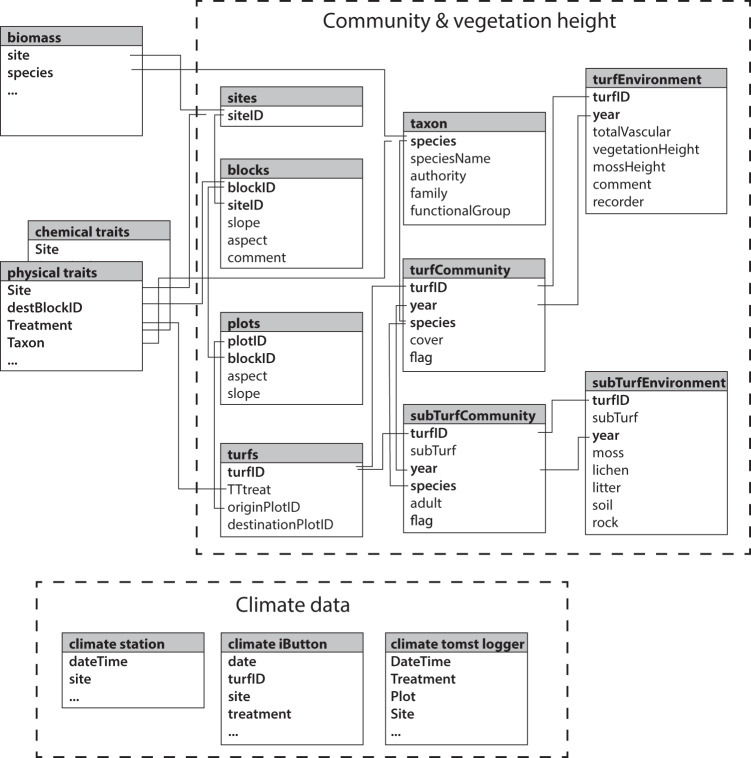
Data structure for the Mt. Gongga plant community, vegetation height and structure (datasets i and ii), biomass (dataset iii), traits (datasets iv) and climate (datasets v-vii) data tables (boxes) and databases (several linked datasets delimitated by hatched lines). Names of individual data tables are given in the grey title area, and variables within tables in the internal lists. Grey lines link variables (bold) that are in common between tables. Links to/from the climate data are not indicated, but can be seen by matching variable names (keys).

This paper reports on data from field experiments on climate change impacts on alpine grasslands of Mt. Gongga in the Hengduan Mountains, Sichuan Province, China conducted from the 2013 growing season onwards. It consists of plant community and climate observations collected from 2012 (pre-treatment year) through 2016, biomass data from 2015, vegetation height from 2013 and plant trait data sampled and analysed in 2015 and 2016. Data outputs consists of seven datasets, the (i) species composition of the experimental plots, (ii) vegetation height and structure of the experimental plots, (iii) biomass harvested in an additional set of control plots at each site, (iv) plant functional traits of individuals sampled from the sites along the gradient and from the experiments, (v) climate and environmental data of the sites and/or treatments, (vi) iButton temperature data from controls and OTCs at all sites, and (vii) TomsT logger temperature and soil moisture data from controls and OTCs at the High alpine site (Table 2). The structure and relationship between datasets are described in Fig. 2. These data were checked and cleaned according to the procedures described under Technical validation before final data files and associated metadata were produced.

**Table 2:** Overview of datasets from the Mt. Gongga climate gradient and experiments.

Dataset	Response variable	Number of data points^a^ and taxa^b^	Temporal range	Predictor variable protocol chapters	Response variable protocol chapters	Citation for clean data, raw data and code
i	Species composition and abundance	9,770^a^; 118^b^	2012–2016	Research sites and climate data; Transplant and OTC experiments	Species identification, taxonomy, and flora; Community composition sampling	Clean data^43^; Raw data^43^; Code^44^
ii	Vegetation height and structure	140^a^	2013	Research sites and climate data	Vegetation height	Clean data^43^; Raw data^43^; Code^44^
iii	Biomass	1,342^a^; 122^b^	2015	Research sites and climate data	Species identification, taxonomy, and flora; Community composition sampling	Clean data^43^; Raw data^43^; Code^44^
iv	Plant functional traits	36,743^a^; 193^b^	2015–2016	Research sites and climate data; Transplant and OTC experiments	Species identification, taxonomy, and flora; Trait sampling and analyses	Clean data^43^; Raw data^43^; Code^44^
v	Climate - stations	1,176,156^a^	2012–2016	Research sites	Air temperature	Clean data^43^; Raw data^43^; Code^44^
vi	Climate - iButtons	444,035^a^	2017	Research sites and OTC experiments	Air, ground and soil temperature	Clean data^43^; Raw data^43^; Code^44^
vii	Climate - TomsT loggers	131,769^a^	2019	Research sites and OTC experiments	Air, ground and soil temperature and soil moisture	Clean data^43^; Raw data^43^; Code^44^

Description and location of the datasets; response variable, number of observations and duration of the data, protocols, final published data and original data and code for extracting data from the primary databases.

The final data files (see Table 2 for an overview) and all raw data, including leaf scans, are available at Open Science Framework (OSF)^43^, and the code necessary to access the raw data and produce these cleaned datasets, along with a readme file that explains the various data cleaning steps, issues, and outcomes, are available in an open GitHub repository, with a versioned copy archived in Zenodo^44^. Note that although parts of these data have previously been made available in connection with earlier publications^29,31,38^, the files reported in the current paper and associated OSF project contain the complete and final datasets. The reader is referred to the code and the detailed coding, data cleaning, and data accuracy comments and the associated raw and cleaned data and metadata tables for detailed information about the data cleaning process.The Usage notes section in this paper summarises the data accuracy and data cleaning procedures, including caveats regarding data quality and our advice on ‘best practice’ data usage.

### Dataset (i): Plant community composition

**Fig. 3:**
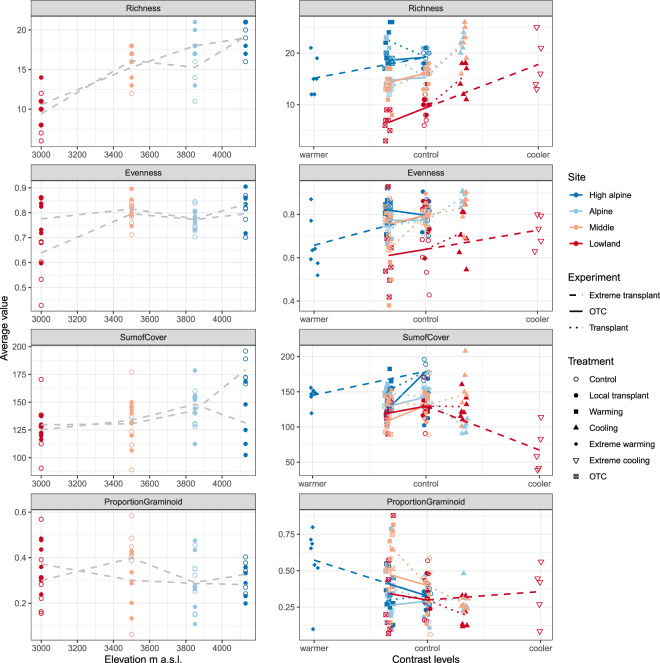
Community characteristics in response to the 1100 m elevation gradient (left-hand panels) and climate change experiments (right-hand panels) in Mt. Gongga. Change in species richness, evenness, sum of covers, and proportion of graminoids with elevation (left hand plots), and in response to climate change treatments by OTCs and transplants (right hand plots). The treatments are expressed as contrasts from controls, moderate and extreme cooling and warming (one or three ‘steps’ to the left and right of the control level), respectively. Different colours indicate the sites, High alpine, Alpine, Middle and Lowland, and symbols show the treatments; control, local transplant, OTC and extreme transplant. Gradients are indicated by grey lines and experiments by lines coloured by origin site. OTCs are indicated by solid lines, extreme transplants by stapled lines, and moderate cooling a warming transplants by dotted lines (see legend).

The plot-level plant community dataset (available in^43^, where the final cleaned dataset is found in the ‘Community’ folder) has a total of 118 taxa and 9,770 observations (taxa x plots x years) (Tables 2 and 3). This dataset is located in the turfCommunity table in the vegetation database (Fig. 2). For details on the clean dataset and the code to clean and extract these data from the raw data, see Table 2. Mean species richness per plot and year (mean ± SE) is 14.6 ± 0.17 species overall and is decreasing from 16.5 ± 0.27 at the High alpine site via 16.2 ± 0.27 at the Alpine site and 15.8 ± 0.27 at the Middle site to 9.9 ± 0.27 at the Lowland site (Fig. 3). In contrast, only weak patterns with elevation exist for evenness, sum of covers, and proportion graminoids along the gradient (Fig. 3).

**Table 3:** Data dictionary for the plant community data from Mt. Gongga (dataset i).

Variable name	Variable type	Variable range or levels	How measured	Units/formats/treatment level coding
originSiteID	factor	[site]	defined	High alpine (H), Alpine (A), Middle (M), Lowland (L)
originBlockID	factor	[site] × [block]	defined	A1 – L7
turfID (same as originPlotID)	factor	[site] × [block] × [treatment]	defined	A1-C – L7-OTC
destBlockID	factor	[site] × [block]	defined	A1 – L7
destSiteID	factor	[site]	defined	High alpine (H), Alpine (A), Middle (M), Lowland (L)
TTtreat	factor	[treatment code]	defined	control, local control (local), warming (warm1), extremeWarming (warm3), cooling (cool1), extremeCooling (cool3), OTC (OTC)
year	date	2012–2016		AC
Species	factor	Short latin species name	Identified	3 digit genus and 3 digit species
cover	numeric	0.01–98	Visually estimated	%
flag	text	Cover corrections and imputations		Comments on cover corrections and imputations
speciesName	text	latin species name	identified	full species name

Data dictionary with column descriptions for dataset i – the plant community composition data file from 140 control and climate change experimental plots at four sites along the 1100 m elevational gradient in Mt. Gongga, China.

The experimental treatments strongly influence all of these community metrics (Fig. 3). Richness and evenness generally increase with cooling and decrease with warming, but with context-dependencies with respect to the type of treatment (OTC vs. transplant and warming vs cooling), position along the gradient, and the severity of the transplant treatment (transplant vs extreme transplant). Sum of covers and proportion graminoids are even more variable.

An NMDS ordination of the full plant community dataset (not shown, but code available in^44^) illustrate shifts in the plant community composition with elevation and experimental treatments. Transplant communities generally converge towards their respective destination warmer-climate or colder-climate communities, with extreme transplant communities changing faster than adjacent-site transplants. OTC communities change slower than the transplant communities change, and they generally do not converge towards their destination warmer-climate communities. There is also community turnover in the local transplants and controls, but this is not directional over time.

### Dataset (ii): Vegetation height and structure

Vegetation height and other structural variables (available in^43^, where the final cleaned dataset is found in the ‘Community’ folder) are located in the turfEnvironment table in the vegetation database (Tables 2, 4 and Fig. 2). For details on the clean dataset and the code to clean and extract these data from the raw data, see Table 2. Vegetation height after 1 year of treatment increased in the OTCs compared to the control plots by (mean ± SE) 3.01 ± 0.19 cm in the High alpine, 4.21 ± 0.26 cm in the Alpine, 8.58 ± 0.89 cm in the Middle, and 8.22 ± 1.13 cm in the Lowland site (F1,54 = 5.75, P = 0.02) (Table 2, Fig. 3).

**Table 4:** Data dictionary for the vegetation height and structure data from Mt. Gongga (dataset ii).

Variable name	Variable type	Variable range or levels	How measured	Units/formats/treatment level coding
turfID	factor	[site] × [block] × [treatment]	defined	A1-C – L7-OTC
year	date	2012–2016		AC
moss	numeric	0–100	Visually estimated	%
lichen	numeric	0–50	Visually estimated	%
litter	numeric	0–115	Visually estimated	%
soil	numeric	0–80	Visually estimated	%
rock	numeric	0–2	Visually estimated	%
TotalVascular	numeric	20–100	Visually estimated	%
vegetationHeight	numeric	0–61.7	measured	cm
mossHeight	numeric	0–12.0	measured	cm
comment	text			
recorder	text			

Data dictionary with column descriptions for dataset ii – the vegetation height and structure in 2013 data file from 140 control and climate change experimental plots at four sites along the 1100 m altitudinal gradient in Mt. Gongga, China.

### Dataset (iii): Above-ground biomass

The above-ground biomass dataset (available in^43^, where the final cleaned dataset is found in the ‘Biomass’ folder) is based on a sample of 20 plots per site, except from the High alpine site, where we sampled only 13 plots (Tables 2 and 5). Biomass is reported per species per 50 cm × 50 cm plot.  Total plot biomass summed over all species increases toward lower elevations along the gradient, with a peak in the Middle site (F3,69 = 19.02, P < 0.001; Table 1). For details on the clean dataset and the code to clean and extract these data from the raw data, see Table 2.

**Table 5:** Data dictionary for the biomass data from Mt. Gongga (dataset iii).

Variable name	Variable type	Variable range or levels	How measured	Units/formats/treatment level coding
site	factor	[site]	Defined	High alpine (H), Alpine (A), Middle (M), Lowland (L)
plot	factor	plot number	Defined	1–20
biomass	numeric	0.004–86.2	Weighed	g
cover	numeric	0.1–80	Visually estimated	%
height	numeric	0.3–46.2	Weighed	cm
n	numeric	number of individuals	Counted	count
speciesName	factor	text	latin species name	identified
authorithy	factor			
genus	factor	text	latin genus	identified
family	factor	text	latin family	identified

Data dictionary with column descriptions for dataset iii – the biomass data file from 73 additional 50 cm × 50 cm control plots at four sites along the 1100 m altitudinal gradient in Mt. Gongga, China.

### Dataset (iv): Plant functional traits

We measured traits for a total of 6,671 leaves from 193 taxa across all sites and treatments, for a total of 36,743 trait observations (Tables 2 and 6).

**Table 6:** Data dictionary for the plant functional traits from Mt. Gongga (dataset iv).

Variable name	Variable type	Variable range or levels	How measured	Units/formats/level coding
^a^Envelope_Name_Corrected	character	[unique code]	defined	
Date	date	August 2015 and 2016	defined	yyyy-mm-dd
Elevation	numeric	3000, 3500, 3850, 4100	gps	m a.s.l
Site	factor	[site]	defined	High alpine (H), Alpine (A), Middle (M), Lowland (L)
destBlockID	factor	[site]x[block]	defined	A1– L7
Treatment	factor	[treatment]	defined	Gradient leaves: (LOCAL)
				Experimental leaves: Control (C), local control (0), warming (1), extremeWarming (3), cooling (2), extremeCooling (4), OTC (OTC)
Taxon	factor	latin name	identified	Full latin name
^a^Individual_number	factor	Five individuals	assigned	1–4
^a^Leaf _number	factor	Five leaves/individual	assigned	1–4
^a^Wet_Mass_g	numeric	0.0004–41.0	weighed	g
^a^Dry_Mass_g	numeric	0.00001–2.87	weighed	g
^a^Leaf_Thickness_1_mm (1–6)	numeric	0.002–2.2	measured	mm
^a^Leaf_Thickness_mm_ave (mean)				
^a^Leaf Area_cm2	numeric	0.01–523.0	measured	cm^2^
^a^SLA_cm2_g	numeric	11.7–499.0	calculated	cm^2^/g
^a^LDMC	numeric	0.009–0.986	calculated	g/g
^b^StoichLabel	character		defined	
^b^P_percent	numeric	0.0458–0.516	measured	%
^b^C_percent	numeric	36.0–51.2	calculated	%
^b^N_percent	numeric	1.25–6.18	measured	%
^b^CN_ratio	numeric	3.00–37.4	measured	
^b2^dN15_permil	numeric	−5.78–11.7	measured	‰
^b^dC13_permil	numeric	(−33.5)–(−25.6)	measured	‰
^a^WetFlag	text	Flags on wet mass		
^a^DryFlag	text	Flags on dry mass		
^a^ThickFlag	text	Flags on thickness		
^a^AreaFlag	text	Flags on leaf area		
^a^GeneralFlag	text	General flags		
^a^allComments	text	General comments		

Data dictionary with column descriptions for dataset iv – the plant traits data file from the site and experimental plot sampling from four sites along the 1100 m altitudinal gradient in Mt. Gongga, China. Note that transplants to adjacent sites are coded as ‘warming’ or ‘cooling’ whereas transplants between the gradient endpoints are coded as ‘extremeWarming’ or ‘extremeCooling’ in the database.

NOTE: ^a^Leaf structural/physical trait data set. ^b^Chemical trait dataset.

From all these leaves, leaf physical or structural traits (leaf area, leaf thickness, specific leaf area [SLA], and leaf dry matter content [LDMC]) were measured (available in^43^, where the final cleaned dataset is found in the ‘Traits’ folder). Specifically, the site-level leaf sampling outside the experimental plots resulted in 4,894 leaves and 193 taxa, with a variable number of observations per site (Lowland = 1,387; Middle = 1,436; Alpine = 1,065; High alpine = 1,006). In addition, the within-treatment level sampling resulted in 1,777 leaves; with 14–404 individuals per species.

Because many specimens had very small leaves, it was necessary to merge some individuals to obtain enough material for the chemical traits. A subset of 576 such combined leaf samples (265 from the site-level samples of sites along the gradient; 311 from the within-treatment level sampling) were thus used to assess leaf chemical or nutrient (Carbon [C], Nitrogen [N], Phosphorus, C:N ratio) and isotope (C, N) traits (available in^43^, where the final cleaned dataset is found in the ‘Traits’ folder).

**Fig. 4:**
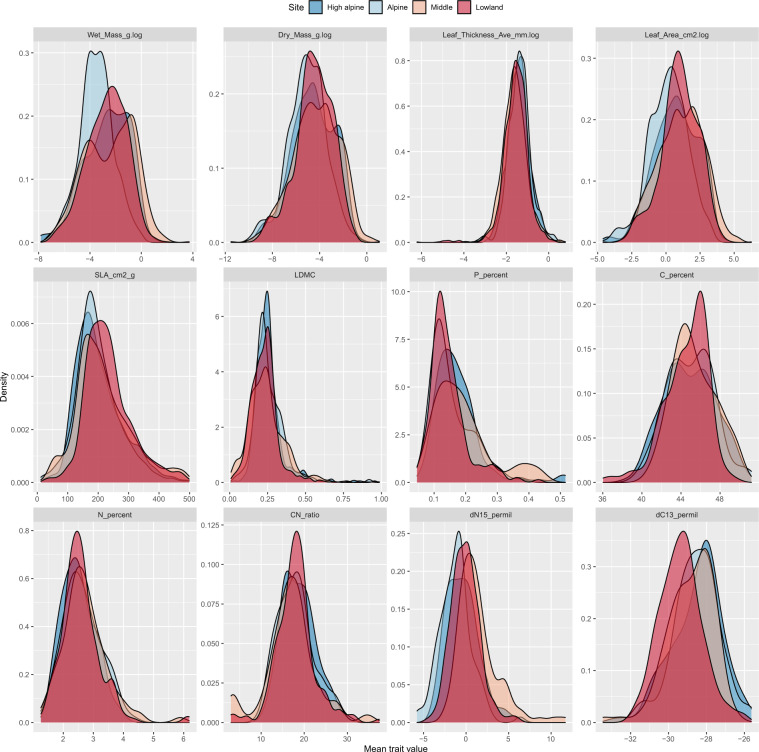
Trait distributions from four sites along the Mt. Gongga elevation gradient. Distributions of trait data (unweighted values) based on all sampled leaves (gradient and experimental plot sampling) from the four sites along the 1100 m altitudinal gradient in Mt. Gongga, Sichuan, China.

Because of leaf merging and hence differences in resolution, the leaf and chemical traits are provided in two data sets. Unweighted trait distributions of all data per site are provided in Fig. 4. The trait data covered between 57% and 85% of the species present in the plant community, based on sum of covers from the control plots, and between 97% and 100% of the biomass at each site comprised of genera with trait data (calculations based on datasets i and iii).

### Datasets (v–vii): Climate

**Fig. 5:**
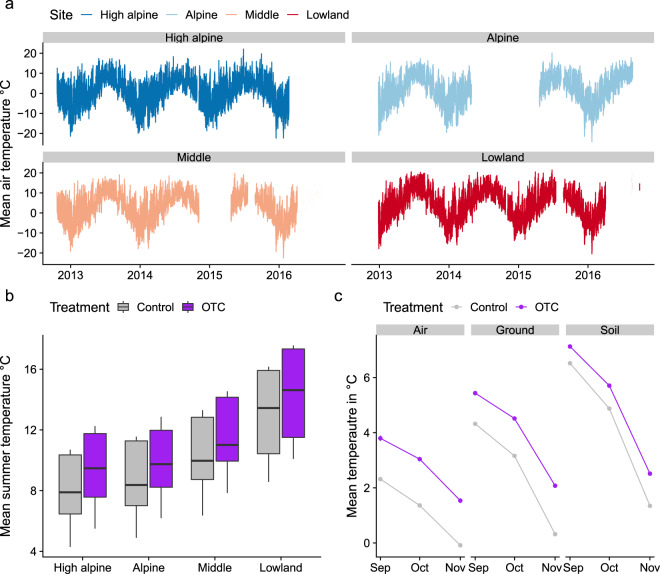
Temperature data from the sites along the 1100 m elevation gradient and open top chambers at Mt. Gongga. (**a**) Continuous (10 minute interval) temperature at 2 m above ground from the climate stations (dataset v), (**b**) mean temperature during the growing season (June-September) at 30 cm above ground inside and outside OTCs at each site (dataset vi), and (**c**) monthly mean temperatures at 15 cm above ground, at ground level, and 6 cm below ground in the OTCs at the High alpine site (dataset vii).

Climate station data was collected, but is of variable quality due to setup and operational errors. To mitigate this, we have extracted partial data for parameters and periods with more reliable data, and augmented these with short-term data from iButton and TomsT loggers. We thus report on climate station air temperature at 2 m at each site along the gradient between December 2012 and October 2016 (available in^43^, where the final cleaned dataset is found in the ‘Climate’ folder). This dataset contains 1,176,156 observations. Average annual temperature in the four sites was 4.73 °C in the Lowland, 3.92 °C in the Middle, 2.05 °C in the Alpine and 1.5 °C in the High alpine site (Dataset v, Fig. 5, Table 7). From the lowest to the highest elevation sites, the mean growing season temperature (June-August) decreased from 12.0 and 6.7 °C, growing degree days above 5 °C decreased from 112–181, and number of frost days (days below 0 °C) increased from 90 to 165 (see Table 1). Unfortunately, the quality of the climate data does not allow us to calculate snow duration (specifically, soil and ground layer temperature loggers were installed for this purpose, but the data are compromised). Snow depth was not measured. We do note, however, that snow cover is not constant, but scattered and highly variable during the winter season at all four sites (Y. Yang, personal observation).

**Table 7:** Data dictionary for air temperature data from the weather station from Mt. Gongga (dataset v).

Variable name	Variable type	Variable range or levels	How measured	Units/formats/level coding
dateTime	date	[yyyy-mm-dd hh:mm_ss]	logged	
Site	factor	[site]	defined	High alpine (H), Alpine (A), Middle (M), Lowland (L)
Air temperature (Tair)	numeric		logged	°C

Data dictionary with column descriptions for dataset v – the weather station data from four sites along the 1100 m altitudinal gradient in Mt. Gongga, China.

The iButton dataset (available in^43^, where the final cleaned dataset is found in the ‘Climate’ folder) contains temperature data through the growing season 2017 (Dataset vi, Tables 2 and 8). This dataset contains 444,035 observations with ca. 140,000 observations for each depth. The warmed plots in the transplants and OTC were on average 0.48–1.74 °C and 0.7–1.58 °C warmer than the control plots (dataset vi, Fig. 5, Table 8).

**Table 8:** Data dictionary for air and soil temperature data from iButtons from Mt. Gongga (dataset vi).

Variable name	Variable type	Variable range or levels	How measured	Units/formats/level coding
Date	date	[yyyy-mm-dd hh:mm_ss]	logged	
Temperature (value)	numeric		logged	°C
turfID	factor	[site]x[block]x[treatment]	defined	A1-C – L7-OTC
Site	factor	[site]	defined	High alpine (H), Alpine (A), Middle (M), Lowland (L)
Treatment	Factor	[treatment code]	Defined	Control (C), OTC
depth	factor	[code]	defined	air (30 cm), ground (0 cm), soil (−5 cm)

Data dictionary with column descriptions for dataset vi – the TomsT logger data from Open Top Chambers (OTCs) and control plots at four sites along the 1100 m altitudinal gradient in Mt. Gongga, China.

From September 2019, air and soil temperature and soil moisture was measured by TomsT loggers in three locations inside and outside the OTC in the High alpine site (available in^43^, where the final cleaned dataset is found in the ‘Climate’ folder). The OTCs had on average higher temperatures compared to the control plots: the difference for air, ground and soil temperature was (mean ± SE) 1.6 ± 0.06 °C, 1.4 ± 0.05 °C and 0.9 ± 0.03 °C, respectively (Dataset vii, Fig. 5, Table 9).

**Table 9:** Data dictionary for air and soil temperature data from TomsT loggers from Mt. Gongga (dataset vii).

Variable name	Variable type	Variable range or levels	How measured	Units/formats/level coding
File	text		defined	File name
DateTime	date	[yyyy-mm-dd hh:mm_ss]	logged	
VolumetricMoisture	numeric	0.0381–0.485	logged	
Treatment	Factor	[treatment code]	defined	Control, OTC
Plot	factor	1–4	defined	
Variable	factor		defined	AitTemperature (30 cm), GroundTemperature (0 cm), SoilTemperature (−6 cm)
Temperature	numeric	−42.3	logged	°C
YearMonth	date	[yyyy-mm-dd]	defined	15^th^ of each month

Data dictionary with column descriptions for dataset vii – the iButton data from transplanted plots, Open Top Chambers (OTCs) and control plots at four sites along the 1100 m altitudinal gradient in Mt. Gongga, China.

## Technical Validation

### Taxonomic validation

During the 5-year data collection period a number of different people were involved, which increases the risk of observation errors in the data set. In particular, species can be misidentified (i.e., sterile graminoids) in the community or traits data or might be overlooked in one of the community censuses. These errors will result in pseudo-turnover in the plant community data and may affect trait distributions. To detect and correct such errors in the community data, we compared each recorded species in each subplot over the 5-year time-series. We used the subplot data to assign unidentified or missing species to an existing identified species in that subplot if it was likely (e.g., from field notes, description or a given lay or generic name) that this could be the correct ID, and if there was a record of that species in the subplot in the previous and following year. Further, we re-estimated species covers in cases where cover was either missing or clearly too low or high to be realistic when comparing with the total sum of covers and covers of the same species in adjacent years in the time-series. We replaced such erroneous values with the mean cover from the previous and following year. We did such re-estimations for a total of 48 occasions (c. 1% of the whole 5-year dataset). The data-checking code and outcomes for these various procedures is documented in^44^.

Both trait and community taxonomy was checked and corrected against TNRS^37^. A full species list of all identified species across datasets is also available in the OSF repository (see below). In addition, there are 10 unidentified taxa in the plant community (dataset i), 29 in the biomass (dataset iii) and 16 in the traits (dataset iv). Note that unknown taxa were not harmonized between the datasets, so that *Genus* sp1 in the trait dataset is not necessarily the same as *Genus* sp1 in the biomass dataset.

### Experimental treatment effects and side-effects

Generally, OTCs increase mean daily air temperature by ca. 1.5 °C^24^. Unfortunately, due to extensive climate logger failure in our experiment we do not have continuous time-series of measurements from the OTCs, but we do have measurements from shorter periods in the growing season in 2017 and in the fall of 2019 indicating that our OTC have similar warming effects to those reported elsewhere (Fig. 5, Datasets vi, vii).

For the transplant experiment, one plot at each block per site was transplanted to the corresponding block of the site at the lower elevation and one plot at the higher elevation, representing a c. 1.75 °C warming or cooling of the summer temperature (i.e., reflecting the temperature difference between each site, dataset v). The extreme transplants were subjected to a 5.3 °C warming or cooling, respectively (i.e., the temperature difference between the High alpine and Lowland sites, dataset v).

Analysis of the local transplant and the untouched control plots across all years show that there were no differences between them in any of the measured predictor or response variables, and thus there were no unwanted biotic or abiotic effects of the turf cutting and transplanting, as also shown in a similar transplant experiment elsewhere using the same approach^26^.

### Trait data validation

Trait data were thoroughly checked and validated as follows. First, we checked and corrected missing or erroneous sample identifications in one or more of the measurements against field notes and notes on the leaf envelopes. Second, unrealistically high or low values of one or more trait values were checked and corrected against the lab and field notes for typing errors. Any remaining samples with apparent measurement errors that resulted in unrealistic trait values were removed. This was done for leaves with clearly erroneous leaf area values (empty scans, double scans, blank areas within the leaf perimeter, dirt or other non-leaf objects on scans, wrong match between scan and leaf ID, etc.), leaf dry matter values higher than 1 g/g, leaves with specific leaf area values less than 5 cm^2^/g or greater than 500 cm^2^/g and leaf nitrogen values higher than 6.4% (see the code and associated readme file^44^ for details). The nitrogen cut-off values was chosen based on global leaf nitrogen values in the Botanical Information and Ecology Network datasets^30^ for the genera in our study. We further plotted the data (e.g., wet mass vs dry mass) and checked for outliers. Some leaves were very small, and dry weights may be approaching the lower limits of weight sensitivity of the balances. In these cases, the data were removed. The data checking code and outcomes for these various procuedures is available and documented in the code and associated readme file^44^.

## Usage Notes

To properly use these data, be aware that (a) in the community data, graminoids are generally not identified to the species level, due to difficulty in identifying sterile graminoids in this regional flora, etc. (see above). The graminoids thus have lower taxonomic resolution than the forbs. (b) Also in the community data, cover values may be prone to observer error due to different observers between years. (c) In the traits data, we have followed what we consider best practice for data quality, and filtered out what we consider unreliable data, e.g., dry mass for very small leaves approaching the limits for balance accuracy, leaf area in the case of clearly erroneous scans, clearly unrealistic measurements (see above). The procedures and consequences are detailed in the code and associated readme file. Users who might prefer a stricter or more inclusive data handling strategy should check the flags in the raw data sets and adjust the data cleaning accordingly. (f) Note that unidentified taxa are not harmonized across datasets. See the code and associated readme file^44^ for our suggested data cleaning and checking procedures that result in producing what we consider the clean and ‘best practice’ final datasets (found in separate folders per dataset in^43^), and the various ‘Flag’ and ‘Comment’ columns in the different dataset tables (Tables 3, 4 and 6) that indicate additional specific data points or leaves (i.e., data rows) that could be removed to create even more robust datasets.

## Data Availability

The code used for checking, cleaning and analysing the data is available in the open GitHuB repository “https://github.com/Plant-Functional-Trait-Course/PFTC_1_2_China”, of which a versioned copy is available at Zenodo^44^. There is also a link to the code from the published dataset^43^.
